# Supplementary figures and images for: Feature selection based on neighborhood rough sets and Gini index
Source: PeerJ Comput Sci. 2023 Dec 12;9:e1711. doi: 10.7717/peerj-cs.1711 (PMC10773927; doi:10.7717/peerj-cs.1711)

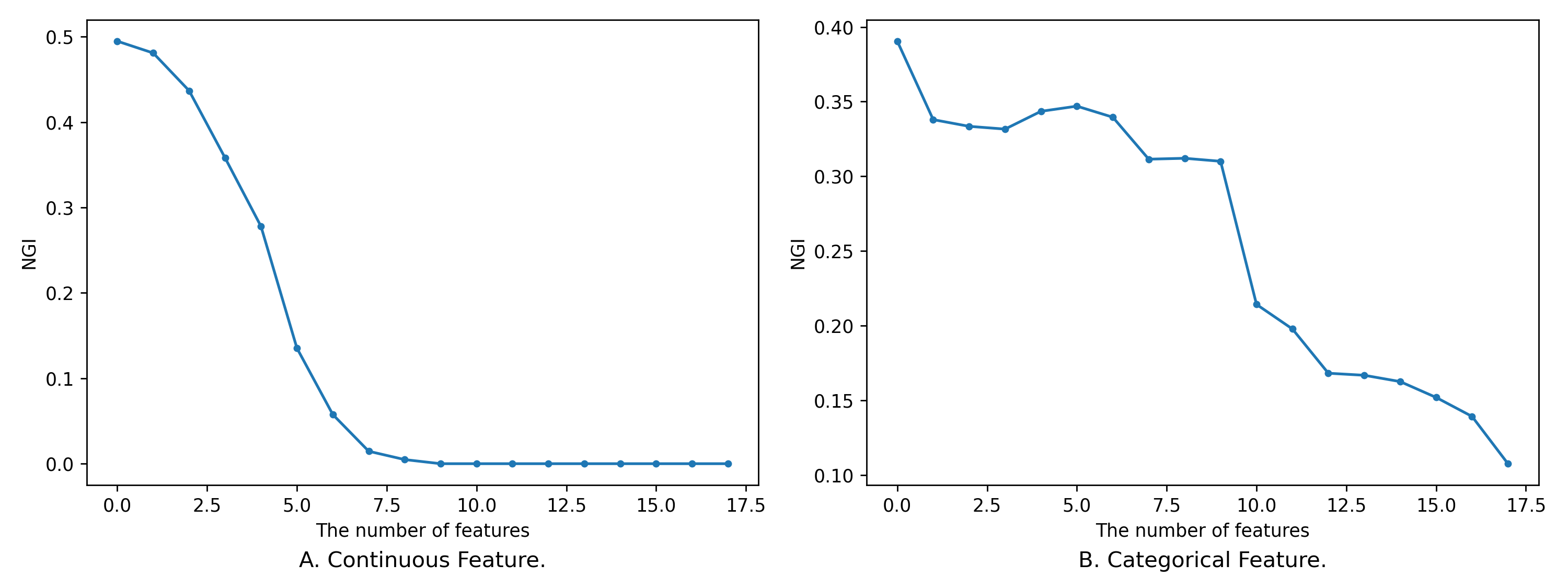

Supplement: Supplemental Information 1 [file peerj-cs-09-1711-s001.zip › upload code/picture/Figure 1.png]

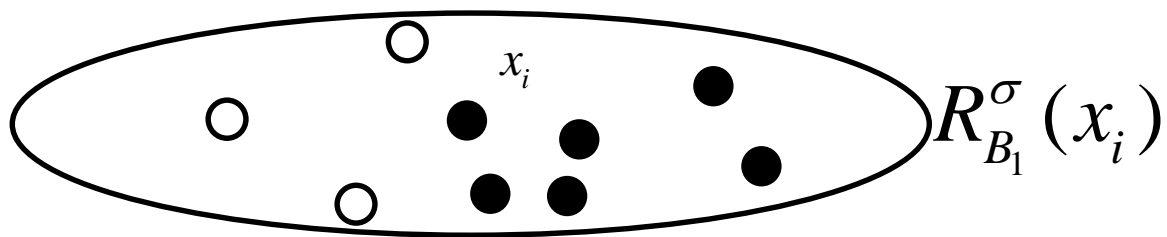

Supplement: Supplemental Information 1 [file peerj-cs-09-1711-s001.zip › upload code/picture/Figure 2.pdf]

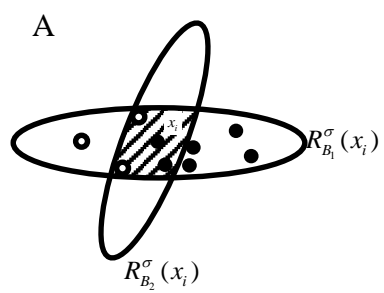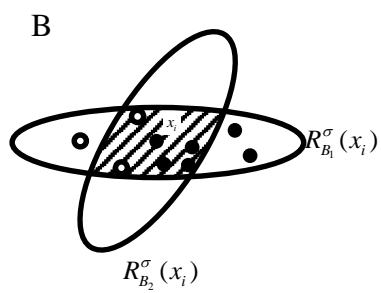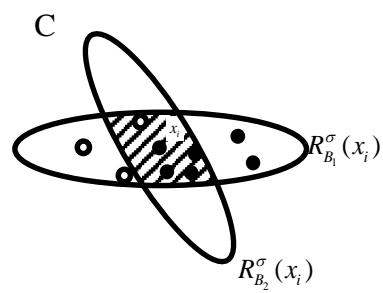

Supplement: Supplemental Information 1 [file peerj-cs-09-1711-s001.zip › upload code/picture/Figure 3.pdf]

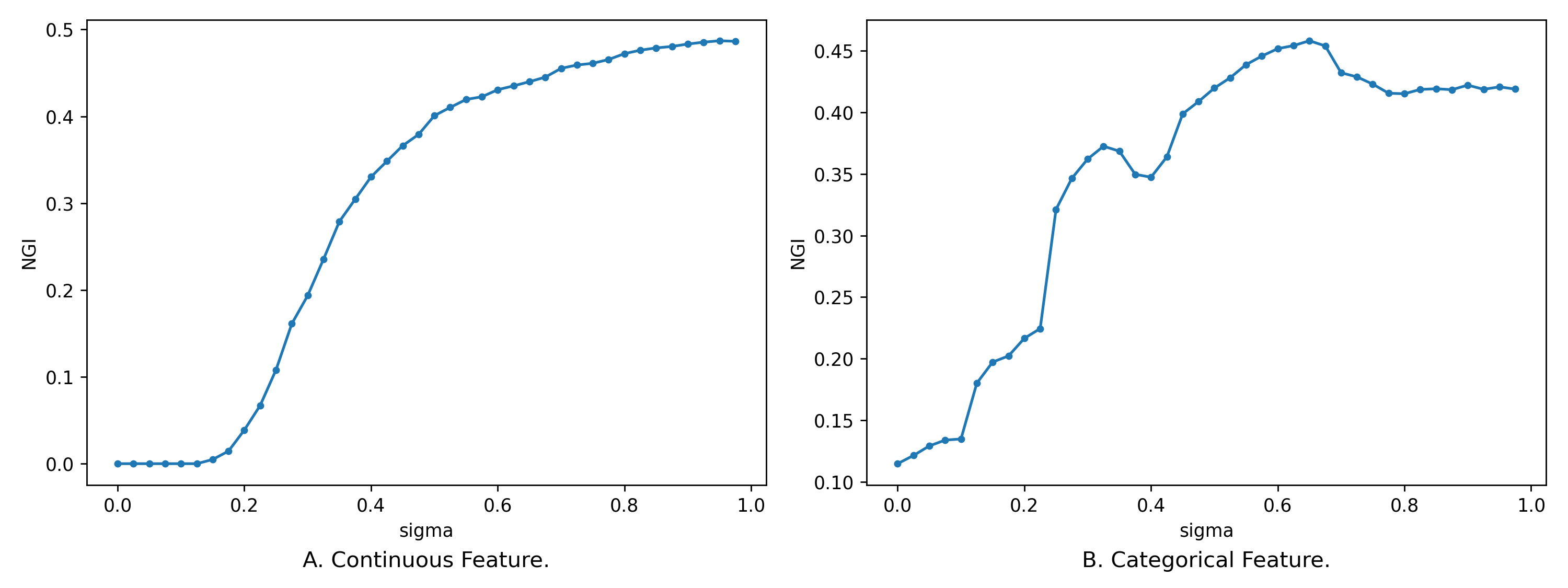

Supplement: Supplemental Information 1 [file peerj-cs-09-1711-s001.zip › upload code/picture/Figure 4.png]

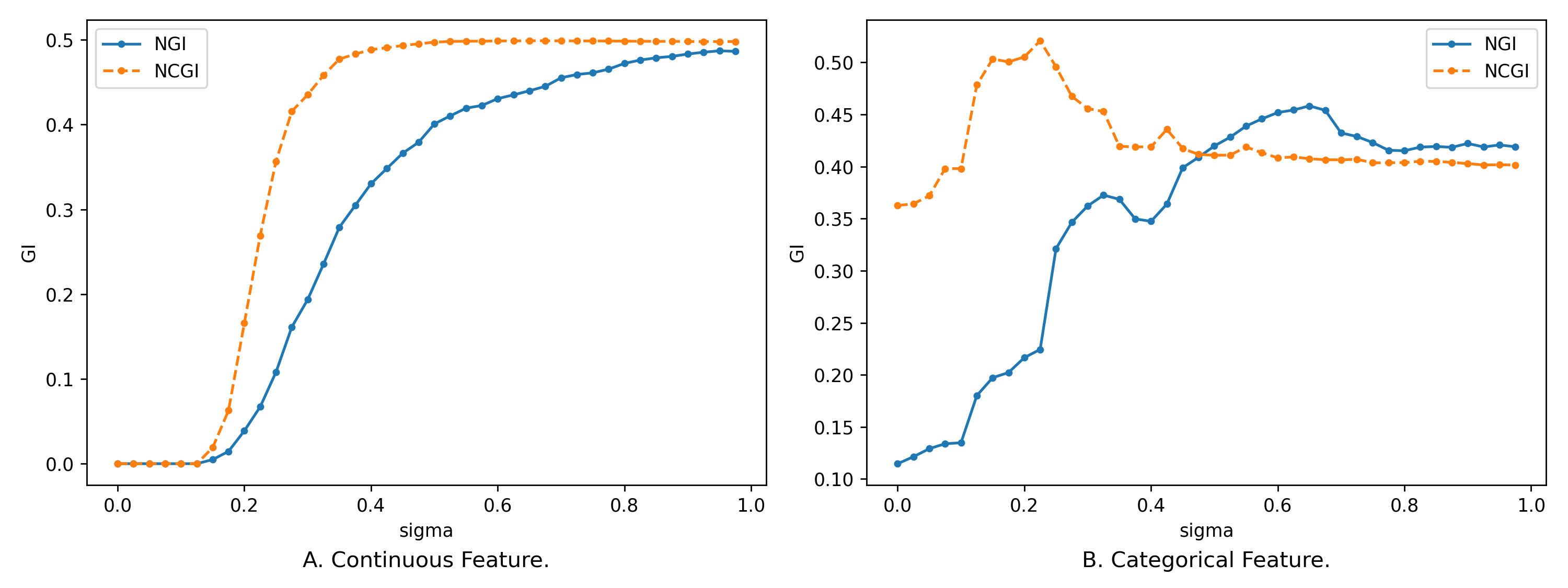

Supplement: Supplemental Information 1 [file peerj-cs-09-1711-s001.zip › upload code/picture/Figure 5.png]

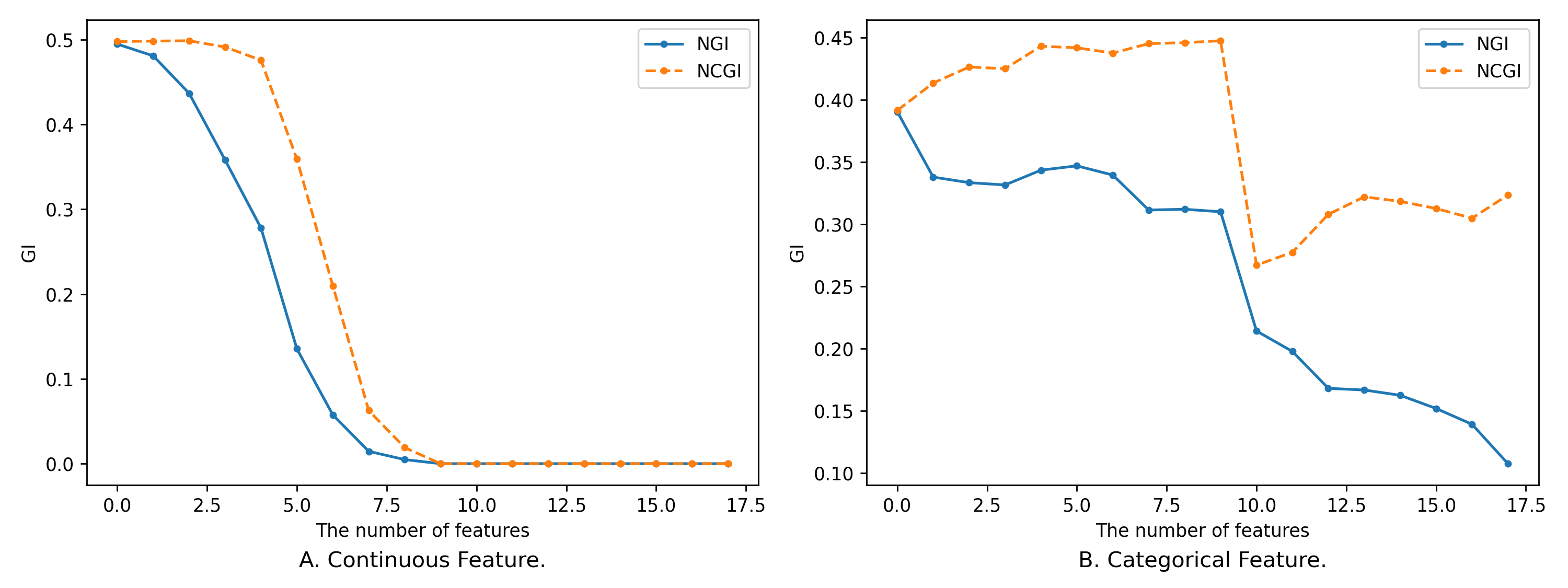

Supplement: Supplemental Information 1 [file peerj-cs-09-1711-s001.zip › upload code/picture/Figure 6.png]

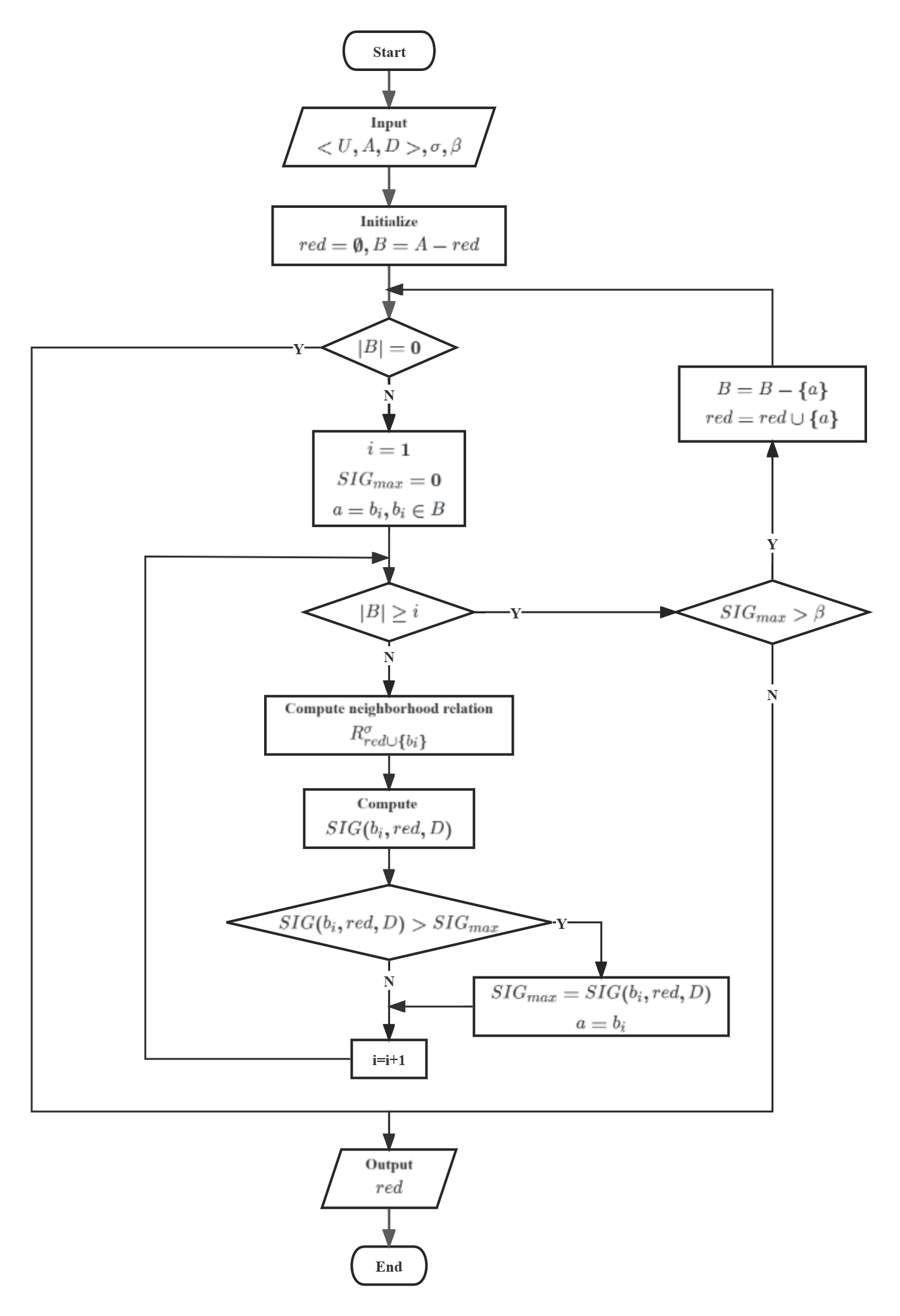

Supplement: Supplemental Information 1 [file peerj-cs-09-1711-s001.zip › upload code/picture/Figure 7.png]

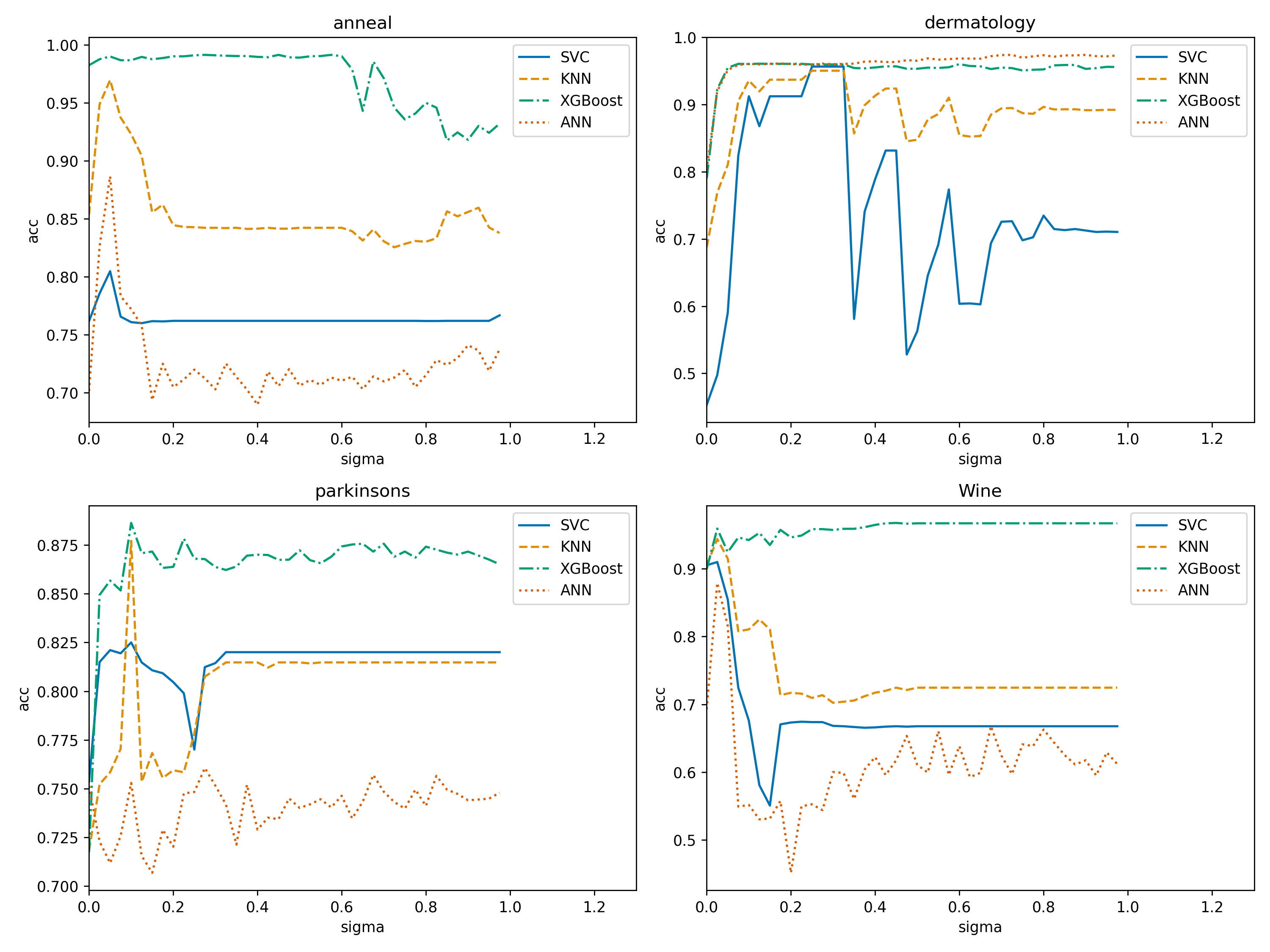

Supplement: Supplemental Information 1 [file peerj-cs-09-1711-s001.zip › upload code/picture/Figure 8.png]
